# Associated Factors of Cholelithiasis among Younger Children with Sickle Cell Disease at the National Reference Center for Sickle Cell Disease in Brazzaville, Congo

**DOI:** 10.1155/2023/8887981

**Published:** 2023-09-27

**Authors:** Firmine Olivia Galiba Atipo Tsiba, Clément Pacha Mikia, Jennifer Armandine Elira Samba, Jade Vanessa Nziengui Mboumba, Félix Malanda, Clausina Mikolele Ahoui, Alexis Elira Dokekias

**Affiliations:** ^1^National Reference Center for Sickle Cell Disease “Antoinette Sassou Nguesso”, Brazzaville, Congo; ^2^Marien Ngouabi University, Brazzaville, Congo; ^3^Gastroenterology Department, Teaching Hospital of Brazzaville, Brazzaville, Congo

## Abstract

**Introduction:**

Chronic hemolysis predisposes sickle cell patients to the development of gallstones. Their frequency increases with age, but they may appear early in young children. In the absence of management, they expose the patient to complications that can hinder the quality of life and sometimes even death. This survey aimed to identify the associated factors of the occurrence of cholelithiasis.

**Materials and Methods:**

It was a case-control study carried out between January 2017 and June 2022 at the National Reference Center for Sickle Cell Disease (SCD) “Antoinette Sassou N'guesso” in Brazzaville. It concerned 37 children with cholelithiasis. Sociodemographic (socioeconomic status and diet) and clinical (body mass index, frequency of vasoocclusive crises and hospitalization for vasoocclusive crises, number of blood transfusion, and chronic complications) as well as hematological examination (type of SCD and blood count in the intercritical period) and hydroxyurea treatment were compared with those of 74 children with no clinical and radiographic signs of cholelithiasis. The chi-squared statistical test and the odds ratio were used for the comparison (*p*  <  0.05).

**Results:**

The average age was 9.70 ± 1.73 years. The 10–12 age group was the most represented (22 cases or 59.45%), followed by 7- to 9-year-olds (12 cases or 32.43%). Three children (8.10%) were 6  years old. The sex ratio was 0.68 *vs.* 1.38. Factors associated with cholelithiasis were low socioeconomic status (83.78% *vs.* 45.95%; IC 95% 1.46−3.89; *p* ≤ 0.001), a higher number of blood transfusions (5.54 ± 1.22 *vs.* 2.46 ± 1.13; IC 95% 1.55−6.70; *p* ≤ 0.001), and irregular systematic monitoring (5.54 ± 1.22 *vs.* 2.46 ± 1.13; IC 95% 1.55−6.70; *p* ≤ 0.001).

**Conclusion:**

A national strategy to facilitate access to care for patients living with sickle cell disease is imperative. Moreover, emphasis should be placed on the prevention and early management of acute complications of SCD.

## 1. Introduction

Sickle cell disease is a genetic disease of hemoglobin (Hb) that causes the synthesis of a modified Hb called Hb S. It is the world's most common hemoglobinopathy, especially in sub-Saharan Africa, where 85% of children affected by the disease are born [1, 2]. In the Congo, homozygous and heterozygous affect, respectively, 1.25% and 25% of the population [3]. In their deoxygenated form, Hb S molecules have the property of polymerizing to form intracellular crystals that deform the red blood cell (RBC), giving it its characteristic sickle shape. The deformed RBC thus loses its elasticity properties necessary to pass through the microcirculation. Stiffening and deformation of the RBC and increased blood viscosity explain the vasoocclusive complications of the disease. In addition, they are destroyed faster than normal RBCs, which further accounts for hemolytic anemia [4].

Chronic hemolysis predisposes sickle cell patients to the development of pigment lithiasis [5, 6]. Its frequency increases with age, up to a quarter [7] to a third [8] of children. In Jamaica, they are under 8 years of age in 12% of cases, and this proportion rises to 23% among 11- to 13-year-olds [9]. LB can appear early in very young children under the age of 5, sometimes as young as 2.5 [8, 10]. In Saudi Arabia, just over a third of the children involved are under the age of 12 (35.9% of cases), with a median diagnostic age of 6.9 ± 3.4 years [11]. In the Congo, LB is the most common chronic complication (40.31%) of sickle cell disease in adults [12]. These data on the child are rare. Moreover, cholecystectomy, known as “prophylactic” meaning performed before any complication, is not always accessible to families because of its cost. As a result, cholelithiasis can be an impediment to a child's quality of life through recurrent abdominal pain or even infections and can affect the prognosis due to complications such as computational migration through the bile duct main, cholecystitis, angiocholitis, or pancreatitis. Special emphasis should, therefore, be placed on its prevention or at least ways of delaying its occurrence. This work aimed to identify the factors associated with the occurrence of LB in young children in Brazzaville.

## 2. Materials and Methods

### 2.1. Study Settings and Design

This was a case-control study comparing sickle cell children under the age of 13. The first group consisted of children with symptomatic or no symptomatic cholelithiasis (cases). The second group consisted of children presenting no clinical signs suggestive of cholelithiasis and presenting a normal abdominal ultrasound at the time of inclusion in the study. The average age of diagnosis of biliary lithiasis frequently reported in the literature is 12. Thus, during our study, we selected children aged 12 and under to identify factors associated with the earlier onset of complications by studying the youngest subjects.

It was conducted over a period of 5 years and 6 months from January 2017 to June 2022. It was set up at the National Reference Center for Sickle Cell Disease “Antoinette Sassou N'guesso” in Brazzaville, which is the country's largest center dedicated to the management of people with the disease since 2017. Patients come from every department in the country. An abdominal ultrasound is prescribed as part of a systematic assessment from the age of 10 years or earlier if there are signs suggesting cholelithiasis. The cases were listed, and then each was matched to two controls based on age. Their data were collected retrospectively from medical records.

### 2.2. Study Variables

The variables studied were sociodemographic (socioeconomic status, diet), clinical (body mass index at diagnosis of cholelithiasis for cases and at inclusion for controls, the average annual frequency of vasoocclusive crisis and hospitalization for vasoocclusive crisis for 3 years before diagnosis of cholelithiasis for cases and 3 years prior to inclusion for controls, and number of blood transfusions since birth and whether or not there are other chronic complications at diagnosis of cholelithiasis for cases and at inclusion for controls), biological (type of sickle cell anemia, intercritical blood count including leukocyte count, Hb rate, mean globular volume, mean corpuscular concentration in Hb, and platelet count), and therapeutic (treatment with hydroxyurea prior to diagnosis of cholelithiasis for cases and prior to inclusion for controls and quality of systematic follow-up).

Drawing on the poverty index considered by the World Bank [13], we considered three socioeconomic statuses:

- Low: anyone in household living on less than $2.15 per day (1300 CFA)
- Middle: anyone in household living on between $2.15 and $4.30 per day
- High: anyone in household living on beyond $4.30 per day (2600 CFA)

We considered the diet to be

- high in fat when the patient consumed at least four times a week one of the following foods or groups of foods: beef/deli, fried foods, peanut (in all its forms), cheese/butter/milk, and legumes
- low in fiber when the patient consumed less than four times a week one of the following foods or groups of foods: vegetables, fruits, and cereals

For blood count, the intercritic phase was defined by a period of at least 4 months characterized by the absence of acute infectious, vasoocclusive, and/or anemic complications. Anemia was considered moderate if Hb was above 6 g/dl and severe if it was below or equal to 6 g/dl. Systematic follow-up was considered regular when the child presented at least once a quarter to the National Reference Center for Sickle Cell Disease; it was considered irregular below 4 annual visits.

### 2.3. Statistical Analyses

SPSS version 25 was used for data analysis. The results of the qualitative variables are presented in absolute values and percentages; those of the quantitative variables are in the form of the mean (standard deviation), minimum, and maximum. The chi-squared statistical tests and the odds ratio were used for the comparison of variables, with a threshold of significance *p* < 0.05 and a 95% confidence interval. The univariate statistical analysis was conducted.

## 3. Results

During the study period, 101 children (aged 0–18 years) were diagnosed with cholelithiasis, 41 of whom were 12 years of age and younger. Thirty-seven complete files were used for this study. The average age of children was 9.70 ± 1.73 years with extremes of 6 and 12 years. The 10–12 age group was the most represented (22 cases or 59.45%) followed by 7–9 years (12 cases or 32.43%). Three children (8.10%) were 6 years old.

The circumstances for the discovery of cholelithiasis were isolated biliary colic (21 cases or 56.76%), followed by cholecystitis (7 cases), systematic ultrasound (6 cases), angiocholitis (2 cases), and biliary peritonitis (1 case). The gallbladder was the primary site of the stones (32 cases *vs.* 5 cases in the main bile duct), and gallstones were most often multiple (86.49% of cases).

The sex ratio was 0.68 in the cases group *vs.* 1.38 in the controls group.

The average frequency of blood transfusions was 5.54 ± 1.22 in the cases group with extremes of 0 and 20. It was 2.46 ± 1.13 with extremes of 0 and 10 in the controls group.

The average vasoocclusive crisis (VOC) and hospitalization for VOC counts were 5.43 ± 1.32 (extremes 0 and 20) and 1.97 ± 0.60 (extremes 0 and 6) in the cases group *vs.* 5.39 ± 1.20 (extremes 0 and 10) and 1.58 ± 0.69 (extremes 1 and 5) in the controls group. A 12-year-old with cholelithiasis also had avascular osteonecrosis of the femoral head. No chronic complications were recorded in the controls.

Table 1 presents the sociodemographic and clinical characteristics of sickle cell children with and without cholelithiasis and their relationship to cholelithiasis at the National Reference Center for Sickle Cell Disease.

The majority of children with low socioeconomic status had a low-fiber diet (19/31) and irregular medical follow-up (17/31). Moreover, five of the six children with an average socioeconomic level had regular medical follow-ups.

Among children with cholelithiasis, 18 (48.65%) were transfused at least 5 times (including 6 children 10 or more times) *vs.* 10 (13.51%) in the controls group, including 1 child at least 10 times. Therapeutically, 12 children (32.43%) were taking hydroxyurea in the cases group *vs.* 25 (33.78%) in the control group. The average duration of treatment was 37 months for cases and 28 months for controls. The main indication was the high frequency of VOC followed by priapism in boys.

Genotype SS was most represented (83.78% *vs.* 71.62%), followed by sickle beta-zero-thalassemia (13.51% *vs.* 10.81%) and sickle beta-plus-thalassemia (2.70% *vs.* 17.57%). All children had moderate anemia. For cases, the mean Hb rate was 6.17 ± 0.44 g/dl *vs.* 6.67 ± 0.27 g/dl for controls.

The average MCV was 76.46 ± 2.14 for cases *vs.* 65.53± for controls.

The average MCHC was 21.42 g/dL for cases *vs.* 28.11 g/dL for controls.

Table 2 shows the type of SCD and baseline biological parameters in sickle cell children with and without cholelithiasis and their relationship to cholelithiasis at the National Reference Center for Sickle Cell Disease.

The average number of platelets was 302 ± 30 for cases *vs.* 327 ± 20 for controls. The average number of WBC was 11499 ± 11 for cases *vs.* 12756 ± 23 for controls.

## 4. Discussion

A very significant strong link has been established between the low socioeconomic status and the occurrence of cholelithiasis. In addition, the majority of children with low socioeconomic status had a low-fiber diet and irregular medical follow-up. These results illustrate the likely link between the socioeconomic level, the type of diet, and the quality of monitoring. The irregularity in the rhythm of routine checks was established as a factor significantly associated with the occurrence of cholelithiasis. This could be the expression of the indirect link between poor adherence to treatment and thus poor preventive and curative management of acute complications of the disease, especially those causing hyperhemolysis. Indeed, in our series, patients who have been transfused more than three times since birth were 3 times more likely to develop cholelithiasis. The same observation was made by Koueta in Burkina Faso [14]. In the USA, a study on the effects of chronic transfusions on abdominal sonographic abnormalities in children with sickle cell anemia reported that gallbladder disease was correlated with older age (*p*=0.002), longer duration of transfusions (*p*=0.034), and higher total bilirubin (*p* ≤ 0.001) [15].

Transfusions performed in children at the National Reference Center for Sickle Cell Disease are mainly punctual and carried out for hyperhaemolytic crises, of which bacterial infections and malaria are the main causes. In this context, patients are generally admitted for clinical signs of severe anemia. More rarely, it is about systematic transfusions performed for the management of chronic complications of sickle cell disease such as stroke, but chronic transfusions are difficult to achieve because of the availability of blood products and their cost. The high incidence and severity of bacterial infections justify prevention efforts through antibiotic prophylaxis and vaccination. Moreover, in areas of high malaria prevalence, special emphasis should be placed on mechanical prevention, including insecticide-treated nets and environmental sanitation.

No significant association was found between VOC frequency and the occurrence of LB. Koueta reported a significant risk (OR = 7.6) when children had at least three VOCs per year [14]. Alhawsawi in Saudi Arabia and Martins in Brazil reported similar results to ours with a higher prevalence of LB in children of phenotype SS compared to S*β*-thalassemias and SC without the difference being significant [11, 16].

In the Adeniyi study in Nigeria, the prevalence of gallstones increased with an increase in the number of crisis in the children, but this was not statistically significant [17]. In France, Kamdem reported that among clinical events, VOC, acute chest syndrome, and febrile episodes significantly increased the risk (*p* ≤ 0.001) [18]. In the literature, VOC frequency is rather a known predictor of ischemic complications, including the avascular osteonecrosis of the femoral head [19].

Da Silva in Portugal identified a statistically significant association between a higher number of hospitalizations (*p* ≤ 0.001), chronic complications of the disease (*p*=0.035), and leukocytes >15 000/*μ*L [20].

In a French study, among the baseline biological parameters, hemoglobin, WBC, neutrophils, platelets, MCV, and bilirubin were not significant factors whereas HbF level (*p*=0.028), reticulocyte count (*p* ≤ 0.001), and LDH (*p*=0.020) significantly increased the risk for gallstones. The multivariate Cox analysis including all the significant risk factors in the univariate analysis retained the deletion of 2 alpha genes (HR = 4.66; 95% CI: 1.11–19.52; *p*=0.035) which decreases the risk, the presence of at least one allele (TA_8_) (HR = 2.26 : 95% CI: 1.07–4.78, *p*=0.032), which increases the risk, and the baseline reticulocytes count per 1 × 10^9^/L increase (HR: 1.001; 1.000–1.002, *p*=0.005), as independent and significant predictive factors for gallstones [18]. Meta-analysis including 34 studies showed that the risk of developing cholelithiasis was significantly associated with lower total hemoglobin level (*p*=0.002), lower hemoglobin F level (*p*=0.003), higher total serum bilirubin level (*p* ≤ 0.001), higher reticulocytes count (*p*=0.007), and UDP-glucuronosyltransferase-1A1 enzyme (UGT1A1) promoter polymorphism [21]. The Olatunya study in Nigeria highlights the contribution of UGT1A1 polymorphisms, a nonglobin genetic factor, to the laboratory and clinical manifestations of young Nigerian SCA patients for the first time. It also shows that children with coinheritance of low UGT1A1 (TA) n affinity genotypes may be at risk of gallstone [22].

There were no significant differences between the Hb SS and Hb S/*β* thalassemia groups. Alhawsawi in Saudi Arabia and Martins in Brazil reported similar results to ours with a higher prevalence of cholelithiasis in children of genotype SS compared to S*β*-thalassemias and SC without the difference being significant [11, 16].

The use of hydroxyurea was not significantly related to the occurrence of cholelithiasis. Martins reported the same [16].

## 5. Conclusion

A relationship was found between a low socioeconomic status, a low-fiber diet, and a higher number of blood transfusions. A national strategy to facilitate access to care for people living with sickle cell disease is imperative. In addition, in our context, where acute hemolytic anemia is primarily caused by malaria and bacterial infections, emphasis must be placed on the prevention and early management of infections.

## Figures and Tables

**Table 1:** Sociodemographic and clinical characteristics of sickle cell children with and without cholelithiasis and their relationship to cholelithiasis at the National Reference Center for Sickle Cell Disease.

Characteristics	Cholelithiasis		Without cholelithiasis		OR	IC 95%	*P*
	*n*	(%)	*n*	(%)			
Gender							
Female	22	59.4	31	41.9			
Male	15	40.6	43	58.1			
Socioeconomic status							
Low	31	83.78	34	45.95	2.38	1.46–3.89	**≤0.001**
Middle	6	16.22	40	54.05	0.07	0.01–0.52	
High	0	—	0	—			
Diet							
High in fat	3	8.11	0	0	0.9	0.79–1.03	0.12
Low in fiber	23	62.16	11	14.86	6.82	0.71–27.18	**≤0.001**
Body mass index							
Normal	28	75.68	42	56.76			
Underweight	6	12.22	17	22.97	0.99	0.83–1.97	
Overweight	0	0.00	1	1.35	1.14	0.17–7.40	0.89
Annual frequency of VOC^*∗*^ in the last 3 years							
Mean (min-max)	5.43 ± 1.32 (0–20)		5.39 ± 1.20 (0–10)				
0–3	15	40.54	20	27.03	0.85	0.60–1.20	0.35
4 and more	22	59.46	54	72.97	1.59	0.58–4.30	
Annual frequency of hospitalization for VOC^*∗*^ in the last 3 years							
Mean (min-max)	1.97 ± 0.60 (0–6)		1.58 ± 0.69 (1–5)				
0–3	30	81.08	64	86.49	0.99	0.88–1.12	0.92
4 and more	7	18.92	10	13.51	1.14	0.08–17.11	
Number of blood transfusion since birth							
Mean (min-max)	5.54 ± 1.22 (0–20)		2.46 ± 1.13 (0–10)				
0–3	13	35.14	57	77.03	0.3	0.13–0.67	**≤0.001**
4 and more	24	64.86	17	22.97	3.22	1.55–6.70	
Hydroxyurea treatment							
Yes	12	32.43	25	33.78	1.14	0.51–2.52	0.75
No	25	67.57	49	66.22			
Quality of systematic follow-up							
Regular	19	51.35	59	79.73	0.54	0.33–0.88	
Irregular	18	48.65	15	20.27	3.41	1.28–9.04	**0.005**

^*∗*^Vasoocclusive crises (pain crises).

**Table 2:** Type of SCD and baseline biological parameters in sickle cell children with and without cholelithiasis and their relationship to cholelithiasis at the National Reference Center for Sickle Cell Disease.

Characteristics	Cholelithiasis		Without cholelithiasis		OR	IC 95%	*P*
	*n*	(%)	*n*	(%)			
Type of SCD							
S/*β* thalassemia^*∗*^	6	16.22	21	28.38	0.57	0.25–1.29	0.16
SS	31	83.78	53	71.62	1.17	0.96–1.43	
SC	0	—	0	—			
Hb (g/dL)							
<6	13	35.14	14	18.92	1.82	0.69–4.74	0.21
≥6	24	64.86	60	81.08	0.8	0.54–1.15	
MCV^*∗∗*^ (FL)							
<100	24	64.86	44	59.46	0.86	0.80–1.47	0.58
≥100	13	35.14	30	40.54	1.09	0.44–1.62	
MCHC^*∗∗∗*^ (g/dL)							
<32	19	51.35	23	31.08	1.26	0.63–2.52	0.81
≥32	18	48.65	51	68.92	0.85	0.53–1.38	
WBC^*∗∗∗∗*^ (/mm^3^)							
<12000	21	56.76	44	59.46	0.67	0.39–1.13	0.11
≥12000	16	43.24	30	40.54	1.7	0.85–3.38	
Platelets (giga/L)							
150–450	31	83.78	56	75.68	1.28	0.89–1.82	0.17
≥450	6	16.22	18	24.32	0.51	0.18–1.41	

^*∗*^Sickle beta-zero-thalassemia (5 cases vs 8 controls) and sickle beta-plus-thalassemia (1 case vs 13 controls). ^*∗∗*^Mean corpuscular volume. ^*∗∗∗*^Mean corpuscular Hb concentration. ^*∗∗∗∗*^White blood cells.

## Data Availability

The data used to support the findings of this study are included within the article.
